# Who Engages and Why It Matters? Describing Participant Engagement in a Serious Game Intervention for Patients With Advanced Cancer

**DOI:** 10.4018/ijgcms.316968

**Published:** 2023

**Authors:** Kai-Lin You, Rebecca K. Delaney, Natalie McKinley, Pat Healy, Teresa H. Thomas

**Affiliations:** University of Pittsburgh, USA; University of Utah, USA; University of Pittsburgh, USA; University of Pittsburgh, USA; University of Pittsburgh, USA

**Keywords:** Advocacy, Cancer, Game Engagement, Gamification, Gamified Intervention, Self-advocacy, Serious Game

## Abstract

While the use and benefits of serious games in health care are increasingly recognized, the impact of individuals’ game engagement remains understudied, limiting the potential for impact. This pilot study aims to describe game engagement and its associations with learning outcomes, sociodemographics, and health factors in women with advanced cancer receiving a 12-week self-advocacy serious game intervention. Game engagement was collected from study tablets and weekly self-reported surveys. Participants’ game engagement was overall high but with large amounts of variation and did not differ by their sociodemographics and health factors. Participants with lower baseline symptom severity were more likely to repeat game scenarios, and those who engaged in all scenarios had higher connected strength post-intervention. Knowing what prevents patients with advanced cancer from engaging in the serious game enlightens ways to refine the gamified interventions. Future research is suggested to evaluate patients’ engagement to deepen understanding of its impacts on learning outcomes.

## INTRODUCTION

### Serious Games in Cancer Care

Serious games are an effective approach to improve illness self-management and are increasingly being recognized for their use and benefits for patients’ health and wellness (Krebs et al., 2019). Compared to conventional interventions, serious games provide interactive and enjoyable experiences for patients to learn more effectively (Wouters et al., 2013) and achieve favorable health outcomes (Sardi et al., 2017), including improved emotional health, better self-management skills, and positive behavioral changes (Charlier et al., 2016; Kim et al., 2018a). Among cancer patients, serious games are also proven to improve their symptoms, adherence, self-management, and mood (Hodgkinson et al., 2007; Kato et al., 2008; Kim et al., 2018b; Loerzel et al., 2020; Wang et al., 2019). Although startup costs for serious games can be substantial, once developed and distributed, they are less costly than interventions requiring trained staff and clinicians (Baranowski et al., 2017).

**Figure 1.**
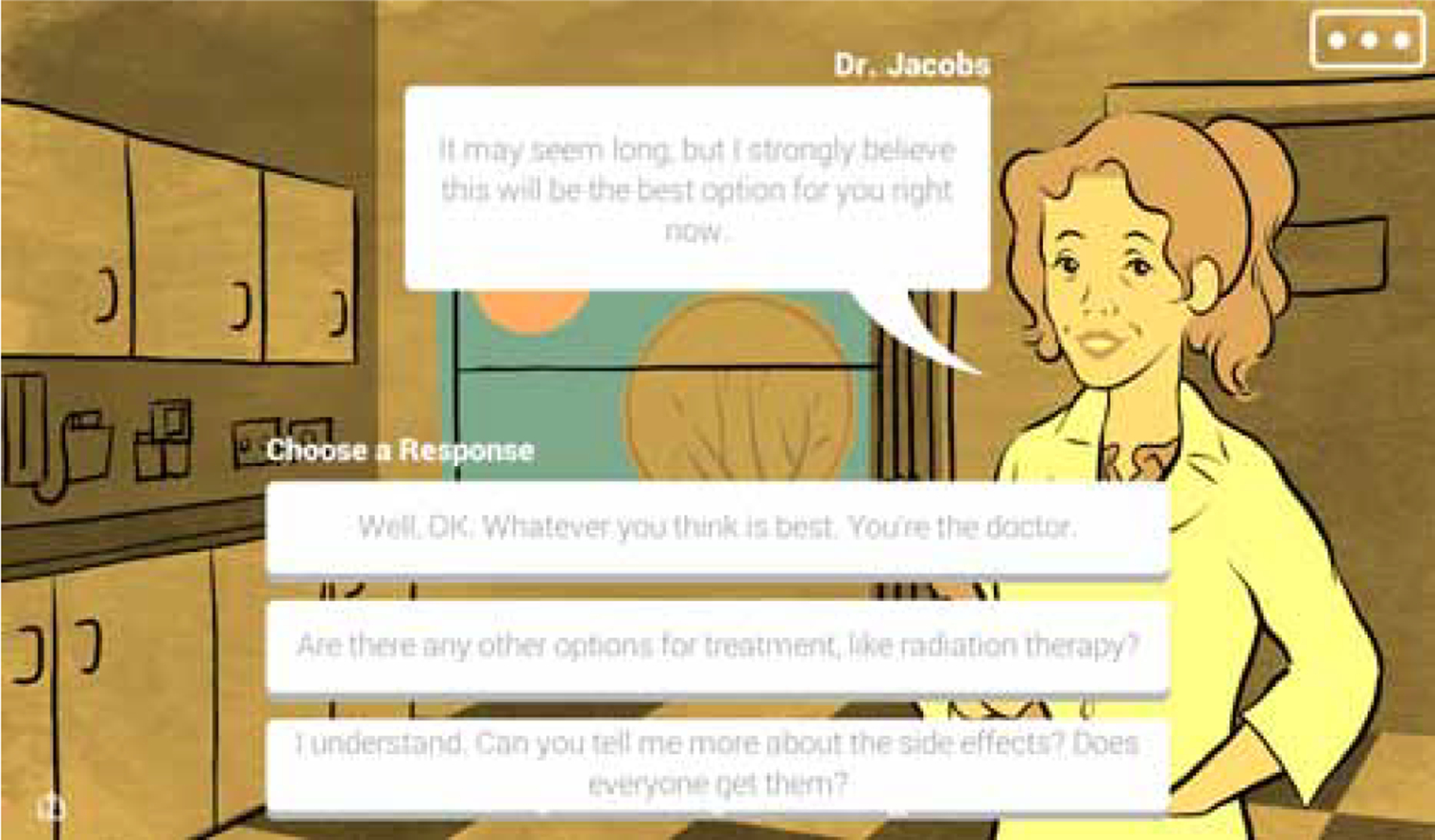
A screenshot of *Strong Together*. The character was meeting with her treating oncologist to discuss her anti-cancer treatment in the clinic. The user is presented with three options on how to respond to the oncologist’s treatment recommendation. Based on this decision, the story progresses with changes based on whether the user’s selection option demonstrates self-advocacy of wanting to know more about the recommended treatment.

One aspect of self-management, self-advocacy, helps patients address challenges related to their health and ensure their care reflects their values and priorities (Thomas et al., 2020a). Self-advocacy skills include being able to (1) make personally meaningful decisions about their cancer care, (2) communicate effectively with healthcare providers, and (3) build strength through connection to others (Hagan et al., 2018). Among women with advanced cancer, self-advocacy is of critical importance for them to cope with the physical, emotional, and decisional challenges of having a serious illness (Keogh, 2014; Wessels et al., 2010). To address a dearth of self-advocacy interventions in cancer, our research team partnered with *Simcoach Games* in Pittsburgh, PA to develop a serious game intervention, *Strong Together*, to help women with advanced cancer learn about self-advocacy skills. In the game, the characters - who are women with advanced cancer - must respond to challenges in their care, and their decisions will decide whether their quality of life improves. The scenarios take place in patients’ familiar places, homes, cancer clinics, or coffee shops, with their significant others, friends, doctors, nurses, or families (Thomas et al., 2019). Figure 1 provides a screenshot of the *Strong Together* intervention.

### Serious Game Engagement

Engagement is an essential indicator in game-based educational studies and was assumed to directly affect learning (Koivisto & Malik, 2021). The definition of game engagement was rarely clearly defined in the studies (Hookham & Nesbitt, 2019). Game engagement is considered ‘the active state of seeking out challenges, which lead to cognitive load’ based on Flow Theory and Cognitive Load Theory (Sharek & Wiebe, 2015). It can be conceptualized as time on tasks, a function of measurable criteria, immersion, presence, flow, absorption, etc. (Hookham & Nesbitt, 2019). While best practices are to integrate knowledge about the intended user (e.g., age, gender, worldview) and situation (e.g., disease types, severity of diseases) throughout the design, implementation, and evaluation, such tailoring of knowledge integration to engage patients is executed inconsistently. When the game mechanics do not reflect users’ traits and demographic characteristics or there is no appropriate feedback regarding users’ performance, users will lose their motivation to engage with a serious game (Sardi et al., 2017). Interviews, indirect observations, and interviews are commonly applied to measure game engagement (Hookham & Nesbitt, 2019). However, there is a lack of shared understanding of how engagement in serious games for health should be evaluated within clinical trials.

The necessary level of users’ engagement to achieve serious games’ learning and behavioral objectives remain empirical questions (Hookham & Nesbitt, 2019; Thomas et al., 2020b). Game engagement can be operationalized by the length of time a user spends on the game (time) (Gorini et al., 2015), the frequency of repeating various game features (depth) (Sharifzadeh et al., 2020), or the completion rates of all game features (breadth) (Simmich et al., 2021). Moreover, evidence is inconsistent on the relationship between the amount of time spent by patients on serious games and intended learning outcomes (Maheu-Cadotte et al., 2021; Pham et al., 2019). To the best of our knowledge, there are no standard forms to assess engagement in serious games or benchmarks to determine the necessary amount of engagement needed to achieve the learning and behavioral objectives of serious games.

This is a pilot study to test the *Strong Together* in women with advanced cancer. Game engagement in this pilot study is operationalized into two ways: completion of all scenarios (breadth) and repetition of any scenarios (depth) and examine their relationship with characteristics of women with advanced cancer: baseline sociodemographic and health-related factors, and 3- and 6-month self-advocacy outcomes. To ensure we catch their use in the intervention period, we apply two approaches to recording the participants’ game engagement, tablet-recorded and self-reported use. Understanding the relationships between various engagement operations and learning outcomes allows researchers to tailor interventions to promote female cancer patients’ engagement and support the equitable distribution of intervention benefits.

The present study aims to add to the serious games for healthcare literature in three ways:

1. Describe two types of serious game engagement, including the number of scenarios engaged and the number of scenarios repeated, within a randomized clinical trial.
2. Determine if the two types of game engagement differed by sociodemographic and health-related characteristics. *Hypothesis 2:* Game engagement will not differ based on participants’ characteristics.
3. Evaluate the association between two types of game engagement and health outcomes. *Hypothesis 3a:* Patients with lower baseline self-advocacy, higher symptom burden, lower quality of life, and lower mood are likely motivated to engage in the serious game to learn self-advocacy skills. *Hypothesis 3b:* At 3- and 6-month, the study’s learning outcome, self-advocacy, will be higher among participants with higher game engagement.

## METHOD

### Sample

Ethical approval for this study was obtained from the University of Pittsburgh Human Research Protection Office (STUDY19050104). This study recruited patients who were: (1) ^3^ 18 years old, (2) female, (3) diagnosed with metastatic breast or advanced gynecologic cancer within the past three months, (4) able to engage in self-care based on an Eastern Cooperative Oncology Group performance status score of £ 2, (5) reported to have at least a 6-month life expectancy according to their oncologist, and (6) English literacy. Research team members recruited eligible participants from cancer clinics within a National Cancer Institute designated Comprehensive Cancer Center in western Pennsylvania. Once participants provided written informed consent and completed baseline study procedures, they were randomized (2:1) to a serious game intervention group (N = 52) or enhanced care as usual group (control group, N = 26). Those in the intervention received a study tablet with the *Strong Together* preloaded.

### Serious Game Intervention

#### Game Introduction

Participants in the intervention group received a 12-week *Strong Together* serious game intervention. The *Strong Together* is designed to teach women with advanced cancer key self-advocacy skills, including (1) making informed decisions about their care, (2) effectively communicating with their healthcare providers, and (3) gaining strength through connection to others (Thomas et al., 2020a). *Strong Together* considered diverse characteristics of patients across subgroups during the game design process. Users follow female characters newly diagnosed with advanced cancer in a fully automated, narrative-based serious game. The game instructions ask users to make decisions to keep the female characters “healthy and strong” as these characters encounter challenges related to their cancer care.

#### Game Scenarios

The serious game is broken down into four scenarios, and each scenario is estimated to take 15 to 20 minutes. Each scenario is similar to an episode of a television series or “chose your own adventure” book. The scenarios include multiple scenes that place the female characters in various challenging situations commonly experienced by women with advanced cancer. For example, participants help the female characters learn to manage uncontrolled symptoms, communicate with an oncologist about treatment decisions, and balance their caregivers’ needs with their own needs. Participants must decide how the character should respond to challenging situations. As participants opt for choices that reflect self-advocacy, the character discovers the positive consequences of self-advocacy behaviors or vice versa. Details of additional game features which reinforce self-advocacy behaviorally and learning objectives have been published elsewhere (Thomas et al., 2019).

#### Retention

Participants received an Amazon Fire tablet with the *Strong Together* pre-loaded along with brief instructions. They were prompted to engage with the serious game about once a week over the 12-week intervention period and encouraged to repeat game scenarios and explore various response options. Participants received a weekly retention email with a brief survey of self-reported game engagement over the past seven days. At the end of the 12-weeks, participants mailed the tablet back to the research team.

### Measures

#### Tablet Engagement Data

Each time a participant opened the *Strong Together*, the *Strong Together* application and the ES File Explorer created a comma-separated values (CSV) file. The CSV file contained the number of specific scenarios the participant played.

#### Self-Report Engagement Data

The weekly retention email asked participants to complete a four-item self-report survey reviewing their use of the *Strong Together* application during the past seven days, including whether they opened the game, time spent on the game, number of scenarios completed, and any repetition of scenarios.

#### Patient-Reported Outcomes

The participants were required to complete surveys at 3- and 6-month after receiving the tablets. The survey included five assessment tools: the Female Self-Advocacy in Cancer Survivorship (FSACS) Scale, Functional Assessment of Cancer Therapy – General (FACT-G), M.D. Anderson Symptom Inventory (MDASI), Hospital Anxiety and Depression Scale (HADS), and Center for Research in Chronic Diseases – Revised survey. The FSACS Scale is a validated 20-item self-report measure of self-advocacy in women with cancer (Hagan et al., 2018). The scoring of each item is from 1 (strongly disagree) to 6 (strongly agree), and the higher the total scores, the better the self-advocacy skills. The FACT-G is a 27-item measure of an individual’s physical, social, emotional, and functional health-related quality of life (Victorson et al., 2008; Webster et al., 2003). The scoring of each item ranges from 0 (not at all) to 4 (very much) and higher sum score suggests better health-related quality of life. The MDASI captures cancer survivors’ symptom burden with two subscales measuring the severity of thirteen common cancer- and treatment-related symptoms and the degree to which symptoms interfere with six daily activities (Cleeland, 2016). Each item is scored on an 11-point scale from 0 (not present/did not interfere) to 10 (as bad as you can imagine/interfered completely). Higher MDASI scores represent higher levels of symptom severity and interference. The 14-item HADS has been validated to assess mood (anxiety and depression symptoms) in cancer patients (Zigmond & Snaith, 1983). Higher HADS scores indicate higher levels of anxiety or depression symptoms, with each item ranging from 0 (not at all) to 3 (always). The Center for Research in Chronic Diseases – Revised survey records individuals’ social and demographic information (Sereika & Engberg, 2006).

## DATA ANALYSIS

Descriptive statistics were used to report participants’ user engagement, including the number of scenarios engaged with, and whether they repeated any scenarios. We used tablet data to examine the two engagement metric distributions: whether the participants complete all scenarios (breadth) and repeat any scenarios (depth).

For aim 1, we used descriptive statistics to describe the distributions of game engagement with frequencies and percentages among the participants. For aim 2, independent t-tests and chi-squared tests were used to determine if the game engagement differed by age, education (years), employment status, racial groups, or income. When there were fewer than five participants, Fisher exact tests were employed. For aim 3, Pearson’s correlation coefficients were used to examine the relationships between game engagement, baseline health-related outcomes, and 3- and 6-month learning outcomes. The independent t-tests were conducted as appropriate to assess whether participants’ game engagement differed in patient-reported outcomes. All analyses were conducted using SPSS Version 28 (IBM Corp.).

## RESULTS

Participant demographic and disease characteristics are summarized in Table 1. The mean age of participants was 59.3 (SD = 13.9) years old. The average number of formal education years was 13.9 (SD = 3.1). Most participants were White (81.6%), followed by Black or African American (15.8%), and Asian (2.6%). Half of the participants were unemployed (55.3%) at the time of enrolling in this study. Most participants (60.5%) were married, and 26.3% were widowed/separated/divorced. The predominant cancer type was metastatic breast cancer (52.6%), followed by advanced ovarian/peritoneal/fallopian tube cancer (36.8%).

### Self-Report Data

**Figure 2.**
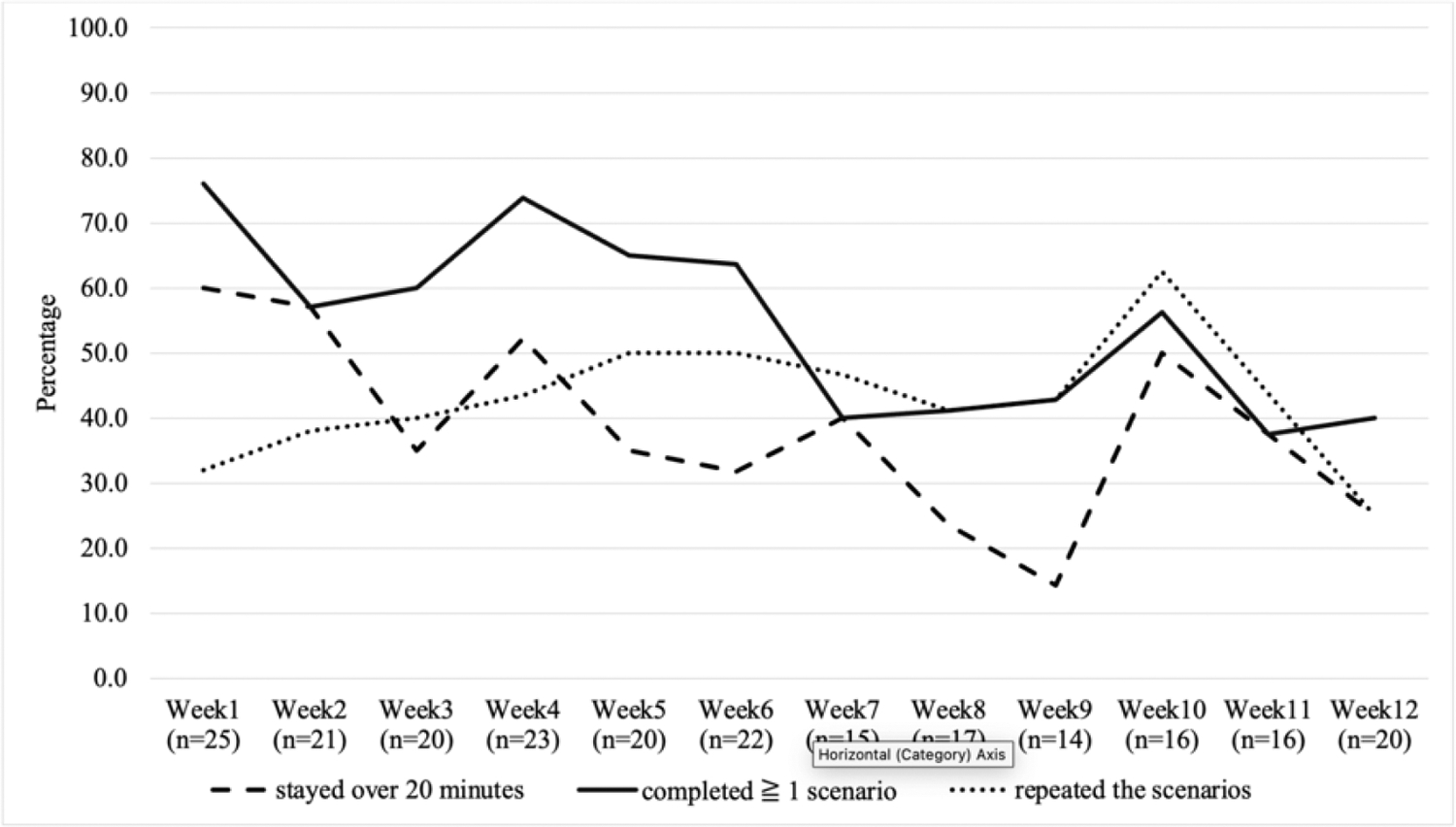
Weekly Trend of Serious Game Engagement Based on Self-Reported Data (N=37)

Figure 2 illustrates weekly game engagement trends from the self-reported data. Each week, between 14 and 25 participants completed the self-report engagement survey. Of those who responded, an average of 66.0% of the participants indicated they had played the game during the past seven days. About 38.5% of the participants who opened the game in the past seven days reporting using the game for over 20 minutes (enough time to complete a scenario). More than half of the participants (54.5%) who opened the game indicated that they completed at least one scenario, and 43.0% of them repeated the scenarios.

### Tablet Data

Of the 52 participants randomized to the intervention, five (9.6%) did not return their tablet, four (7.8%) died, and three (5.8%) withdrew from the study before the 12-week intervention period ended. Two tablets were not included for instances in which technical difficulties provided incomplete tablet data. The total sample with complete data included 38 participants. The average number of plays each patient had was 11.2 times (SD = 16.7; Q1, Q3 = 3.8, 15; range = 0 – 103).

#### Engagement by Sociodemographic and Health-Related Factors

Participant engagement – in two methods of operationalizing it – did not differ by sociodemographic or health-related factors according to independent t-tests and chi-squared test results.

#### Engagement by Scenarios Engaged

The participants were grouped into two groups based on the number of scenarios engaged in the serious game. Participants who engaged in all four scenarios were considered highly engaged users (n = 26/38, 68.4%), and those who engaged in 0 to 3 scenarios were considered relatively lower engaged users (n = 12/38, 31.6%). Highly engaged participants reported significantly higher 3-month self-advocacy skills of connected strength than relatively lower engaged participants (*t* = −2.74, *p* = .01). High versus low engaged participants by scenario engaged did not significantly differ in baseline symptoms, mood, health-related quality of life and self-advocacy skills. Additionally, their game engagement did not differ in 3- and 6-month self-advocacy skills except 3-month connected strength (Table 2).

#### Engagement by Scenario Repetition

The participants were grouped into two groups based on if they had repeated any scenarios in the serious game. Over half of the participants (n = 22/38, 57.9%) repeated at least one of the four scenarios, while 42.1% of the participants (n =16/38) did not. Participants who did not repeat any scenarios reported significantly higher baseline symptom severity than those who repeated at least one scenario (*t* = 2.25, *p* = .03). Participants grouped by scenario repetition did not significantly differ in baseline symptom interference, anxiety, depression, health-related quality of life and self-advocacy skills, and 3-month and 6-month self-advocacy skills (Table 2). The authors reran the analyses after removing one extreme outlier, which did not change the results to confirm the findings.

## DISCUSSION

This study is one of the first and initial steps to provide an in-depth view of serious game engagement in a population of seriously ill patients. The *Strong Together* serious game was designed to be delivered in a naturalistic, non-prescriptive manner and provided with maximum flexibility considering cancer survivors’ heavy disease burdens. Thus, it is unsurprising that game engagement did not differ by the participants’ sociodemographic or health-related factors. The findings of this study underscore that game engagement might be tied to symptom severity in women with advanced cancer. Women with advanced cancer who had lower baseline symptom severity tended to engage and repeat the game scenarios. Regarding the learning outcomes, this study demonstrates that women with advanced cancer who engaged in all four scenarios had significantly better self-advocacy skills of connected strength than those who did not.

As hypothesized, the serious game appears to be equally accessible to people of various ages, education levels, and health statuses which is important for the broad dissemination of the serious game, if efficacious. This result attests to the central concept of employing a user-centered and theoretical approach to develop the serious game intervention in this study (Thomas et al., 2019). The storylines were highly relevant to the challenges that women with advanced cancer encounter. Features within the serious game were designed to be universally acceptable among patients with varying levels of health literacy.

A major finding of this study is that participants who did not repeat as many game scenarios reported to have higher symptom severity at baseline. This study recruited women newly diagnosed with advanced cancer, who were likely to be burdened by cancer-related symptoms at the time of recruitment. The sustainable cancer-related symptoms and coming anti-cancer treatment might decrease the amount of willingness and cognitive effort the participants would voluntarily spend on the educational serious game. The weekly trend of self-reported game engagement indicates that, generally, women with advanced cancer engaged the most in the first few weeks of the intervention period and then moderately decreased after six to seven weeks. This finding aligns with other mHealth studies, which demonstrate that levels of engagement rapidly decline after the first few weeks of intervention use (Pham et al., 2019; Psihogios et al., 2019). Contrary to the hypothesis, participants’ baseline quality of life and mood did not differ by game engagement, indicating these two health-related factors were unlikely to impact participants’ engagement in the serious game intervention. Participants engaging in all four scenarios had significantly better self-advocacy skills of connected strength but not for their other 3- and 6-month self-advocacy skills. Therefore, we suggest that health and priorities among patients with advanced cancer should be taken into consideration. Extending the serious game implementation period might allow cancer patients with serious illnesses to have flexibility in engaging in learning. Subjective measurements, including questionnaires and interviews, are suggested as they provide more specific reasons regarding patients’ engagement levels (Fleming et al., 2016; Hookham & Nesbitt, 2019). More game engagement metrics in terms of time investment and completion rates by the cancer survivors should be tested to understand how they contribute to the learning outcomes.

Little is known about the relationship between game engagement with sociodemographic, health-related, and learning outcomes (Pham et al., 2019). While other pilot studies have demonstrated the positive effects of serious games on cancer patients’ health-related outcomes, including cancer-related fatigue, side effects of chemotherapy, drug adherence, quality of life, and physical function (Kim et al., 2018a; Kim et al., 2018b; Wang et al., 2019), they did not examine the relationships between participants’ engagement and the health outcomes. Examining the impact of engagement on outcomes helps clarify the necessary amount or “dosage” of the serious game to impact major learning and behavioral outcomes. Therefore, this study’s results provide practical implications for researchers to design and test serious game interventions in seriously ill patient populations. The study results suggested that the serious game might need to be tailored considering patients’ pressing health concerns and be tied to their care trajectory. Understanding the reasons leading to various engagement levels in patients with cancer may be helpful for future studies to strengthen their intervention implementation and address the potential barriers of low engagement in patients with advanced cancer. Based on the results of this pilot study, we are developing a large-scale and multi-site randomized controlled trial of the *Strong Together* intervention to determine specific engagement metrics that drive the study outcomes.

### Limitations.

This pilot study has several limitations. This study had a limited sample size and a limited diversity of study participants. The recruited participants reflect the patients in the cancer center we recruited but do not reflect the diversity across the country. This study did not account for all possible factors that could impact participants’ engagement in the serious game, such as technical savvy, motivation, or ability. The Strong Together was designed as a one-user interface for patients’ use and might record caregivers’ engagement as patients’ records since a few patients reported their caregivers had interests and engaged in the serious game. The results were likely impacted by participants’ knowledge that the researchers were reviewing their game engagement through weekly surveys and tablet tracking (e.g., the Hawthorne effect). Finally, the serious game did not automatically record the time spent on the game and the completion rate of each scenario.

We are developing a large-scale randomized controlled trial to address the limitations identified in this pilot study. We will add features to the serious game, including real-time data transmission to a server and various assessments of game engagement (e.g., amount, duration, breadth, and depth) to understand the degree to which participants receive the intervention and the associations with the intended learning outcomes.

## CONCLUSION

Serious games for health are growing and have proved efficacious, yet limited research explores the concept of game engagement with patients’ health and learning outcomes. This study examined multiple factors associated with game engagement among women with advanced cancer to describe how they used the serious game and how engagement differed by their health and learning outcomes. This study provides cues for researchers to tailor serious games for seriously ill patients, which should take their symptom burden into consideration. Future research is suggested to employ objective and subjective measurements to identify what motivates and limits game engagement. Research focusing on serious games for health should continue to explore what dosage is needed to achieve intended learning outcomes. With increased emphasis on identifying how serious game engagement impacts learning outcomes, the empirical science of games for health can more directly and robustly address pressing health problems.

## Figures and Tables

**Table 1.** Participant Characteristics (N=38)

	Mean (SD) or n (%)	
Age, Mean (SD)	59.3	(13.9)
Years of formal education, Mean (SD)	13.9	(3.1)
Household income, n (%)		
0–20k/year	6	(15.8)
20–50k/year	8	(21.1)
50–80k/year	9	(23.7)
80–150k/year	4	(10.5)
>150k/year	4	(10.5)
Unknown	7	(18.4)
Race, n (%)		
White	31	(81.6)
Black/African American	6	(15.8)
Asian	1	(2.6)
Ethnicity, n (%)		
Hispanic/Latina	1	(2.6)
Non-Hispanic/Latina	35	(92.1)
Do not know	2	(5.3)
Employment, n (%)		
Currently employed	14	(36.8)
Retired, but working part/full time	1	(2.6)
Disabled, unable to work	2	(5.3)
Unemployed	21	(55.3)
Marital status, n (%)		
Currently married	23	(60.5)
Living with partner/Significant other	3	(7.9)
Widowed/Separated/Divorced	7	(18.4)
Never married	5	(13.2)
Health insurance*		
Medicare	19	(35.2)
Medicaid/Medical assistance	6	(11.1)
Veterans administration	1	(1.9)
Disability income	3	(5.6)
Private health insurance	25	(46.2)
Cancer type, n (%)		
Metastatic breast	20	(52.6)
Advanced ovarian/peritoneal/fallopian tube	14	(36.8)
Advanced endometrial	3	(7.9)
Advanced vaginal/vulvar	1	(2.7)

Note:

* Some participants have more than one type of healthcare insurance.

**Table 2.** Comparison Tests of Patient-Reported Outcomes by Serious Game Engagement Recorded by Tablets (N=38)

	Score Range	Scenario Engaged		Scenario Repetition	
		0–3 scenarios (n = 12)	All 4 scenarios (n = 26)	Yes (n = 22)	No (n=16)
Baseline Quality of Life	0–108	79 (14.3)	83.7 (11.0)	83.3(9.5)	80.7(15.2)
Baseline Symptoms					
Symptom severity	0–130	43.1(20.9)	32.5 (13.0)	30.7(12.0)*	42.9(19.2)*
Symptom interference	0–60	16.9 (10.4)	19.4 (12.0)	16.7(9.9)	21.3(13.2)
Baseline Mood					
Baseline anxiety	0–21	6.4 (5.6)	4.4 (3.1)	4.0(2.9)	6.6(5.0)
Baseline depression	0–21	3.8 (2.7)	4.3 (3.2)	4.1(3.0)	4.3(3.2)
Baseline Self-Advocacy	20–120	95.3 (8.8)	95.8 (11.6)	95.8(11.3)	95.5(10.1)
Informed decision-making	7–42	35.9 (3.5)	34.5 (4.8)	34.0(4.6)	36.3(4.0)
Effective communication	6–36	29.6 (3.3)	29.7 (3.1)	30.0(3.0)	29.2(3.4)
Connected strength	7–42	29.8 (6.0)	31.7 (6.3)	31.9(5.7)	30.0(6.9)
Self-Advocacy at 3-months	20–120	91.5 (11.3)	99.2 (10.4)	98.0(9.2)	95.9(14.3)
Informed decision-making	6–36	36.3 (4.1)	35.3 (4.8)	35.5(4.4)	35.6(5.1)
Effective communication	6–36	29.3 (5.1)	30.5 (3.4)	30.0(3.1)	30.6(5.0)
Connected strength	7–42	26.0 (7.0)*	33.0 (6.1)*	32.4(5.8)	29.2(8.7)
Self-Advocacy at 6-month	20–120	96.8 (9.7)	101.5 (10.7)	100.7(9.2)	99.6(12.9)
Informed decision-making	7–42	36.4 (4.0)	37.5 (3.6)	37.0(3.7)	37.5(3.9)
Effective communication	6–36	31.2 (4.8)	31.2 (3.1)	30.8(2.8)	31.8(4.5)
Connected strength	7–42	29.1 (8.1)	32.9 (6.7)	32.9(6.2)	30.3(8.7)

Note:

* *p* < .05, *t*-test.
